# Remedies and Their Uses

**Published:** 1907-09-14

**Authors:** 


### Therapeutics.

### Remedies and their Uses.

### Formic Aldehyde and Formic Acid.

The powerful germicidal and antiseptic action of formic aldehyde is well known; this substance is largely used for the sterilisation of instruments and the disinfection of rooms, but its internal administration is rendered difficult by the irritating qualities of its vapour. It has, however, been recently combined with lactose in the form of a tablet for purposes of internal use. This combination, known as formamint, contains 0.01 grain of formic aldehyde in 1 grain of the tabellæ, together with some citric acid as a flavouring agent, and some pepsin-hydrochloric acid in order to ensure the liberation of formic aldehyde. It appears to be clearly shown that after sucking one of these tablets the saliva acquires marked bactericidal properties. Beitzke added a culture of bacillus prodigiosus to ordinary saliva and also to saliva obtained from a person who had sucked formamint. Plates were inoculated from each, and whereas that from normal saliva showed many colonies, that from formamint saliva showed very few, even when the plates were taken at once. One tablet of formamint dissolved in 10 c.c. of water destroys streptococci, pneumococci, the bacillus of typhoid and that of diphtheria in from 5 to 10 minutes (Seifert). Formamint also stimulates the secretion of saliva, but it appears very doubtful if any general effect on the organism is produced by its use. Rheinholdt's experiments on rabbits seem to show that at any rate formamint has no power of disinfecting the bladder or kidneys, though formic aldehyde taken as such, or liberated in the body from such compounds as urotropine, shows marked power in this direction. Thirty-five formamint tablets taken in twenty-four hours failed to produce any bactericidal power in a man's urine.

The results, however, of the local action of formamint on the mouth and pharynx are very satisfactory, and the fact that it appears to have no further effects is of advantage when considered in this connection. In septic conditions of the mouth and throat in children these tablets are very useful. Owing to their pleasant taste they are not rejected by the patients, and by their means such difficult and troublesome procedures as swabbing, gargling, etc. may be obviated. For infants with thrush or stomatitis, a tablet may be powdered and enclosed in a small muslin bag tied on the end of a string. This the child may suck, while the string is held by the nurse; in a few minutes the bag is withdrawn, and the process repeated several times daily. In children of three to five years, half-tablets may be sucked every two hours for a day, after which the acute symptoms of a tonsillitis will probably have subsided. It is important to note that these tablets should be sucked, and not actively crushed by the teeth, as a slow evolution of the active disinfectant principle is required. Adults and children over six years may take the full dose, one every hour or every two hours, according to the severity of the local condition. Besides their use in suppurative tonsillitis, formamint tablets have been employed as a prophylactic when epidemics of sore throat, diphtheria, and scarlet fever are prevalent. Zwillinger reports that the tablets are of value in the anginal stage of scarlet fever, and as an adjuvant to antitoxin in diphtheria. They are prepared by Wulfing and Co., London.

Formic aldehyde itself, the watery solution of which is known as formalin, is usually administered as a urinary antiseptic. A substance named hexamethylene-tetramine liberates the aldehyde in the body, and this excreted by the urine acts in the desired manner. Various trade names have been given to this substance, which shows, at any rate, that there is a considerable use for it among practitioners; urotropine, aminoform, formin, cystamine, cystogen, metramine, uretone, urisol, and vesalvine are all identical chemically.

Experience shows that a dosage of 5 to 10 grains, well diluted, three or more times daily, is usually sufficient; some observers, however, prefer to begin with small doses, rapidly increasing the amount till 45 to 50 grains are taken daily. If any signs of renal or vesical irritation occur they can usually be controlled by omitting the drug for a few days. Formaline itself may be injected intravenously in septicæmia, and formalin enemas (8 drops of the solution in about 1 pint 1-per-cent. salt solution) have appeared to do good in puerperal septicæmia.

*Urogosan* (Merck) is a combination of urotropine and gonosan, another urinary antiseptic. It is sedative as well as astringent, and gives an acid reaction to the urine. It is said to be particularly useful in gonorrhœal cystitis and posterior urethritis, and is prepared in capsules, each containing gonosan grains 5, and hexa-methylene-tetramine grains 2½. Two of these may be ordered thrice daily after food.

*Formicin* (Merck) is an antiseptic prepared by condensation of formic aldehyde and acetamide. It is dispensed as a syrupy liquid, freely miscible with water, with which it breaks up into its constituents. A 5-per-cent. solution may be used instead of iodoform in tuberculous affections of the joints; these are quite painless. Two-per-cent solutions may be used as bladder-irrigations, but stronger solutions cause pain. It has been given as an intravenous injection, but, though apparently harmless, no good results are recorded.

*Formic Acid* in the form of a formate has been recommended by Huchard as a nerve and muscle tonic in pneumonia, influenza, anæmia, neurasthenia, and depressed conditions generally. The dose is 30 to 45 grains daily. These salts are also diuretics; their action begins as early as fifteen minutes after ingestion, and may last two to eight days. The soda salt is deliquescent, and can only be ordered in solution; 15 grains may be given three or four times daily. An elixir containing 15 grains to the tablespoonful is prepared by Roberts and Co.

